# Examinations the optical, mechanical, and shielding properties of Ag_2_O doped B_2_O_3_–Bi_2_O_3_–SrF_2_–Na_2_O glasses for gamma ray shield applications

**DOI:** 10.1038/s41598-022-07450-7

**Published:** 2022-03-03

**Authors:** A. S. Abouhaswa, Mansour Almurayshid, Fahad Almasoud, M. I. Sayyed, K. A. Mahmoud

**Affiliations:** 1 Ural Federal University, Mira St., 19, Yekaterinburg, Russia 62002; 2 King Abdulaziz City for Science and Technology (KACST), Nuclear Science Research Institute (NSRI), Riyadh, 11442 Saudi Arabia; 3 Department of Soil Sciences, College of Food and Agricultural Sciences, King Saud University, Riyadh, 12372 Saudi Arabia; 4 Department of Physics, Faculty of Science, Isra University, Amman, 11622 Jordan; 5 Department of Nuclear Medicine Research, Institute for Research and Medical Consultations, Imam Abdulrahman Bin Faisal University, Ad Dammām, 31441 Saudi Arabia

**Keywords:** Materials science, Physics

## Abstract

A series of five glass samples have a chemical composition of (55-x) B_2_O_3_ + 5 Bi_2_O_3_ + 20SrF_2_ + 20Na_2_O + xAg_2_O with varied doping ratios x = 0, 1, 2, 3, and 4 mol% were fabricated using the melt quenching technique to study the effect of B_2_O_3_ replacement by Ag_2_O on the physical, mechanical, optical and gamma-ray shielding capacity of the fabricated glasses. The Cary 5000 UV–Vis–NIR measured the optical absorption in the wavelength range between 200 and 3000 nm. Based on the measured optical absorption, energy (direct/indirect) bandgap and Urbach energy were calculated. Moreover, the measured samples density, molar volume, packing density, dissociation energy, and mechanical properties for the fabricated glasses were calculated using the concepts of the Makishima-Mackenzie model. In this regard, the microhardness was decreased from 4.070 to 3.931 GPa with raising the Ag_2_O concentration. The effect of B_2_O_3_ replacement on the shielding capacity was also evaluated using the Monte Carlo simulation. The simulation results showed that the replacement of B_2_O_3_ causes a significant increase in the shielding parameters like linear attenuation coefficient and radiation shielding capacity. The best radiation shielding properties were achieved for a glass sample with 4 mol% Ag_2_O compound. Its linear attenuation coefficient varied between 8.091 and 0.134 cm^−1^, raising the gamma photon energy between 0.059 and 2.506 MeV.

## Introduction

Every technological progress has its pros and cons and various risks to the environment and humans. Technological progress provided a paradigm shift in the use of radiation in many fields, especially in the medical sector (whether it is therapeutic or diagnostic), treating serious diseases, and diagnosing patients’ conditions. However, its consequences may lead to unprecedented radiological damages^1–3^. Such damages may result from overexposure to radiation, whether from the occurrence of radiation leakage from the devices used in radiation treatment and diagnosis, from the radioactive materials used in such field, or because of exceeding the permissible limit of radiation in this kind of treatment. Consequently, the abovementioned standpoint should be considered upon designing units of diagnostic and therapeutic radiography, as well as in the research laboratories and other facilities in which radioactive materials and radioisotopes are used^4–7^. Adequate precautions should be taken upon dealing with ionizing radiation, unless it may cause physical damage to workers, patients, or even to visitors of hospitals, as such damages also may be hereditary and appear in future generations. In view of the seriousness of radiation, it should be dealt with properly and safely, as one of the most followed principles for this purpose is to increase the distance between the person and the radioactive source and reduce the time of radiation exposure, as well as the use of radiation protection shields^8–12^.

Nuclear radiation protection shields reduce the radiation exposure of people in places of radiation, and they are called biological shields. They are also used in nuclear reactors and sometimes to protect sensitive electronic devices that may not regularly work in the field of radiation. Such shields are usually used to protect important military equipment and laboratories with electronic devices within the perimeter of nuclear reactors^13–15^. Therefore, it became important to develop new types of nuclear protection shields of radiation, as the same has become an influential part of our daily lives and considered one of the important matters, especially after the great modern scientific progress, which began to focus on the use of radioactive materials and other sources of radiation in the medical and agricultural fields, as well as other scientific fields such as building nuclear research reactors, in addition to the modern applications of energy generation and various aspects of life^16,17^. Determining the thickness of a particular shield or selecting the form of the composition or the quality of the nuclear shield material to protect against a certain type or types of radiation is the basis for studying nuclear radiation shields to reduce the radiation dose to the specified and acceptable level and bring it to the lowest levels allowed for professionals or the public from the people. The shield's thickness and its form of the composition or the quality of its material are the basis for studying nuclear radiation shields to reduce the radiation dose to the specified and acceptable level and provide its lowest levels allowed for the professionals or the public.

In recent times, researchers studied the possibility of using alloys, concrete, ceramics, and glass as effective shields of radiation protection^18–22^. Glass is considered one of the most important materials that researchers have been interested in developing as radiation protection shields because of its ease of preparation, its cheap cost, ease to control its form, shape, and density by changing the oxides of its elements, as well as it is a non-toxic, eco-friendly material and a transparent material that allows the light to penetrate through, which encourages the use of glass in the windows of the walls of the radiology rooms^23–25^. There are various types of glass formations. The B_2_O_3_ glass has received great attention from researchers and nuclear materials engineers due to the distinct physical and chemical properties of this type of glass, such as its low viscosity, high transparency of visible light, low melting point, its low cost compared to other types of glass such as TeO_2_ glass^26^. However, there is a major defect of such kind of glass, as its density is relatively low due to the low density of B_2_O_3_, which reduces the possibility of using this glass in radiation protection shields. The low density of this type of glass can be overcome by using heavy metal oxides, which increase the density of the glass and improve its various physical properties. Many researchers have attempted to study the gamma rays' shielding parameters of different types of glass. We mention here some previous works that used B_2_O_3_ glass and some heavy metal oxides. Asadi and Hosseini^27^ used the Monte Carlo method and studied the radiation shielding properties of B_2_O_3_–Bi_2_O_3_–ZnO–Li_2_O glass samples. The researchers also studied the effect of changing the percentage of both ZnO and Li_2_O on the radiative attenuation properties at energy levels ranging from 200 to 1500 keV. By using the XCOM program, they were able to assess the accuracy of the simulation results, as the results showed that the error rate in the values calculated using MCNP and XCOM is less than 4 at most energy levels. Aboud et al.^28^ studied the radiation protection properties of B_2_O_3_ glass, which contains heavy metal oxides such as PbO and Bi_2_O_3_, as they were able to study the effect of changing the concentration of PbO from 0 to 25 mol% on the radiation attenuation coefficients. Results obtained by the researchers show that there is a possibility of using glass as radiation protection shields. Ibrahim et al.^29^ prepared a set of zinc bismo-borate glasses containing RE and calculated the radiation attenuation parameters using Phy-X/PSD program. Researchers concluded that glass has better radiation shielding properties at low energies. However, the glass's ability to attenuate radiation decreases upon increasing the photon energy. Marltan et al.^30^ used simulation techniques (by utilizing MCNPX code) and were able to examine the effect of both BaO and Bi_2_O_3_ on glass samples that contain B_2_O_3_. Researchers confirmed that the greater the percentage of heavy metal oxides in the glass, the better the glass has the better properties in terms of providing adequate radiation protection. Al-Harbi et al.^31^ were able to examine the effects of the four oxides SrO/TeO_2_/PbO/Bi_2_O_3_ on attenuation of radiation properties for the glass containing B_2_O_3_. The researchers have found that the use of all previous heavy oxides improves the ability of glass to absorb photons, but Bi_2_O_3_ gives better results than the other three heavy oxides. This illustrates the importance of using Bi_2_O_3_ in the manufacture of glass instead of lead to give better results and to get rid of the toxic effects of lead and thus obtain safe and effective glass in radioactive prevention. To complement the great efforts made recently, the researchers in this work have developed a new glass system containing the following oxides: B_2_O_3_, Bi_2_O_3_, SrF_2,_ Na_2_O, and Ag_2_O. They made a comprehensive study of the prepared glass's structural, optical, and radiation shielding properties.

The present work aims to study the impact replacement of B_2_O_3_ by Ag_2_O in a new fabricated glass series consisting of B_2_O_3_–Bi_2_O_3_–SrF_2_–Na_2_O–Ag_2_O. Thus, some of the physical and mechanical properties were analyzed based on the Makishima–Mackenzie model. Furthermore, the optical absorption is determined experimentally. Also, the ability of the fabricated glasses to attenuate the gamma photons with an energy range varied between 0.224 and 2.506 MeV was evaluated.

## Materials and methods

### Fabrication and characterization

Using the well-known melt quenching procedure, five glass samples with nominal compositions (55-x) B_2_O_3_ + 5 Bi_2_O_3_ + 20SrF_2_ + 20Na_2_O + xAg_2_O with varied doping ratios x = 0, 1, 2, 3, and 4 mol% were manufactured. Powders of boron oxide (B_2_O_3_), bismuth oxide (Bi_2_O_3_), strontium fluoride (SrF_2_), sodium oxide (Na_2_O), and silver oxide (Ag_2_O) were carefully mixed to obtain a homogeneous mixture of the glass samples. The mixtures were then put into a porcelain crucible and heated for 30 min at 1000 °C before being abruptly formed into a stainless-steel pattern to form the glass samples discs. Finally, the melting prepared glass samples were quenched in the mold and annealed at 300 °C for 2 h before cooling to room temperature.

After the samples were annealed, the density of the fabricated glasses at room temperature was measured using Archimedes’ method, and the dipping fluid is toluene, which has a density of 0.86 g/cm^3^.

Based on the measured density, calculated molar mass, ionic radius (R), and the dissociation energy for the fabricated glasses’ constituting compounds, the mechanical properties, including the mechanical moduli (Young, shear, bulk, and Longitudinal) and the glass micro-hardness were calculated using the theoretical concepts of Makishima–Makinze (M–M) model^32,33^.

In order to measure the optical absorption of the fabricated samples, the glass samples were firstly polished. After that, the Cary 5000 UV–Vis–NIR double beam spectrophotometer was applied to measure the optical absorption of the polished samples in the wavelength range varied between 200 and 3000 nm.

### The shielding capacity evaluation

**Figure 1:**
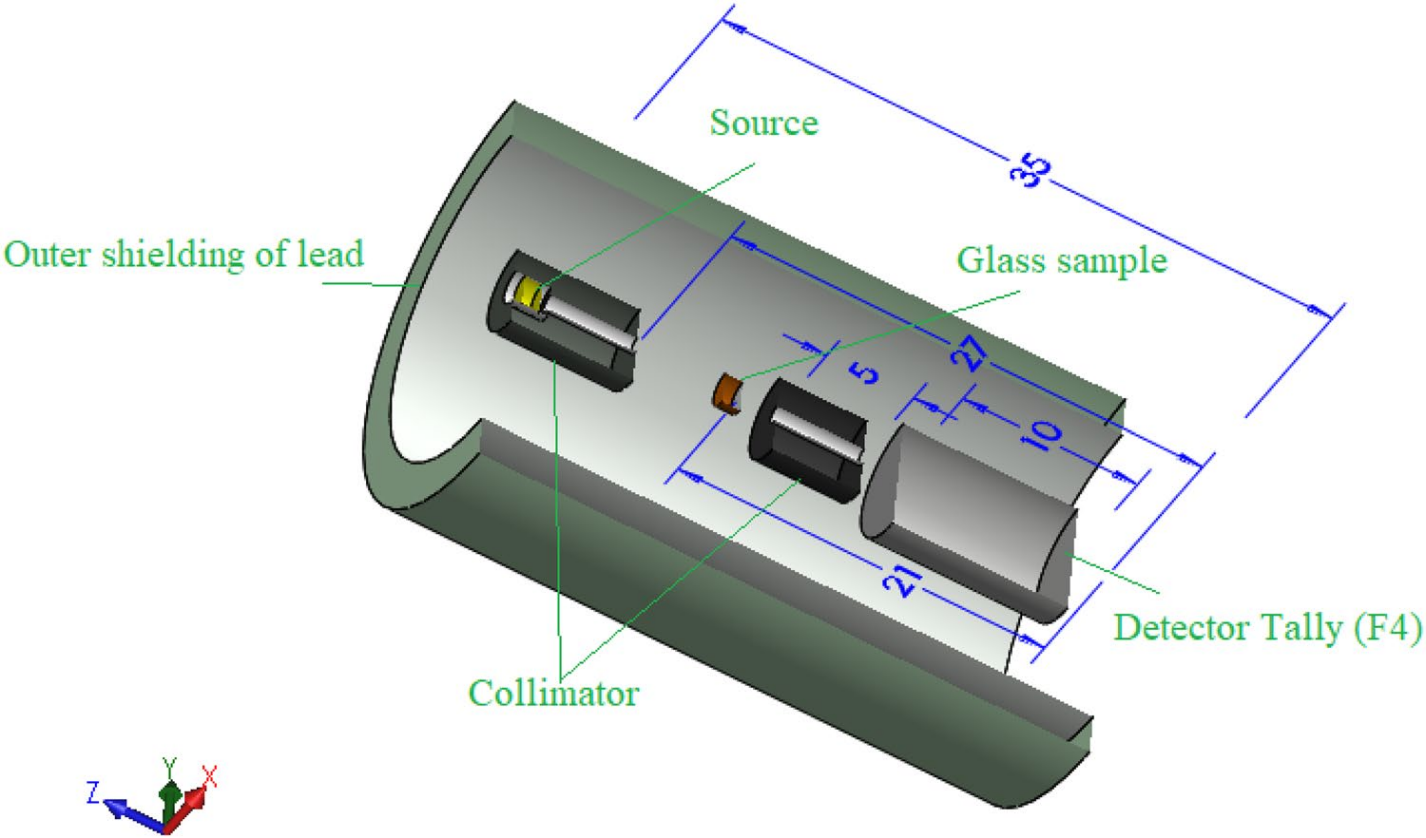
A 3D representation for the MC geometry components.

The evaluation of the shielding parameters was performed utilizing the Monte Carlo (MC) simulation technique in the energy interval between 0.059 and 2.506 MeV. As previously illustrated in many articles, an input file containing all information about the source, detector, sample, and geometry should be created to evaluate the shielding properties using the MC simulation^34,35^. In this input file, the source is considered to emit a photon flux with energies varied between 0.059 and 2.506 MeV along the Z direction. It is placed on the center of the geometry at the origin (0, 0, 0). The sample was placed between two collimators at a distance of 9 cm from the detector while it was far from the source by 10 cm. The chemical composition, density, molar mass, and molar volume are illustrated in Table 1. The tally used in the present study is F4 in order to be calculated inside the fabricated sample. All dimensions and geometry components are illustrated in Fig. 1.

**Table 1:** The chemical composition,density and molar volume of the fabricated BBSNAg glass samples.

Sample	Chemical composition mol%					Density g/cm^3^	Molar volume (cm^3^/mol)
	B_2_O_3_	Bi_2_O_3_	SrF_2_	Na_2_O	Ag_2_O		
BBSNAg0	55	5	20	20	0	3.246	30.527
BBSNAg1	54	5	20	20	1	3.275	30.753
BBSNAg2	53	5	20	20	2	3.311	30.908
BBSNAg3	52	5	20	20	3	3.362	30.925
BBSNAg4	51	5	20	20	4	3.413	30.936

## Results and discussion

### Density and molar volume

**Figure 2:**
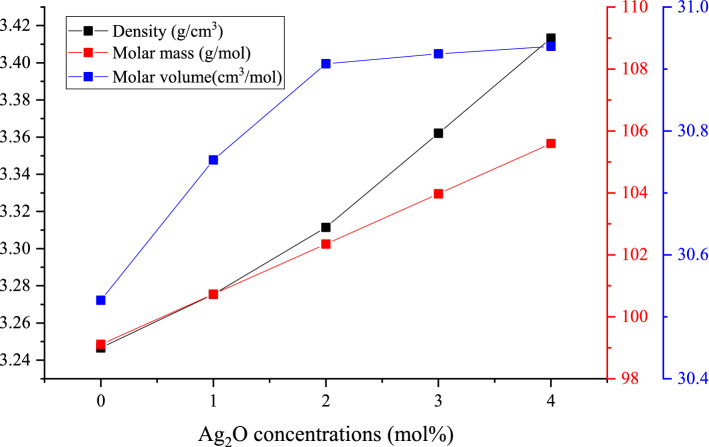
The variation of glass density, molar mass, and molar volume versus the doped Ag_2_O ratio.

The material density is considered one of the most important physical parameters. The determination of the glass density helps to understand the variations that occur in the glass structure due to dopant insertion. Figure 2 illustrates an increase in the fabricated glasses’ density, molar volume, and molar mass with increasing the Ag_2_O doping ratio. Moreover, the fabricated glasses' density increased between 3.247 and 3.413 g/cm^3^ when the Ag_2_O dopant concentrations were increased between 0 and 4 mol%, respectively. Also, the molar mass increases between 99.108 and 105.592 g/mol, with increasing the Ag_2_O doping ratio between 0 and 4 mol%. This increase is related to doping the investigated Bi_2_O_3_–Na_2_O–Sr_2_F–B_2_O_3_ by a dense compound where the Ag_2_O compound has a density of 7.14 g/cm^3^ and a molar mass of 231.375 g/mol. The molar volume of the BBSNAg glasses can describe the glass network structure and the unit cell arrangement. Figure 2 shows that the molar volume of the fabricated glass increases between 30.527 and 30.936 cm^3^/mol, which can be attributed to the homogeneity and opening up the network structure. Also, increasing the molar volume affects the strengthening of the fabricated glasses' chemical bond, which significantly affects the mechanical properties of the fabricated glass samples^36^.

### Optical properties

**Figure 3:**
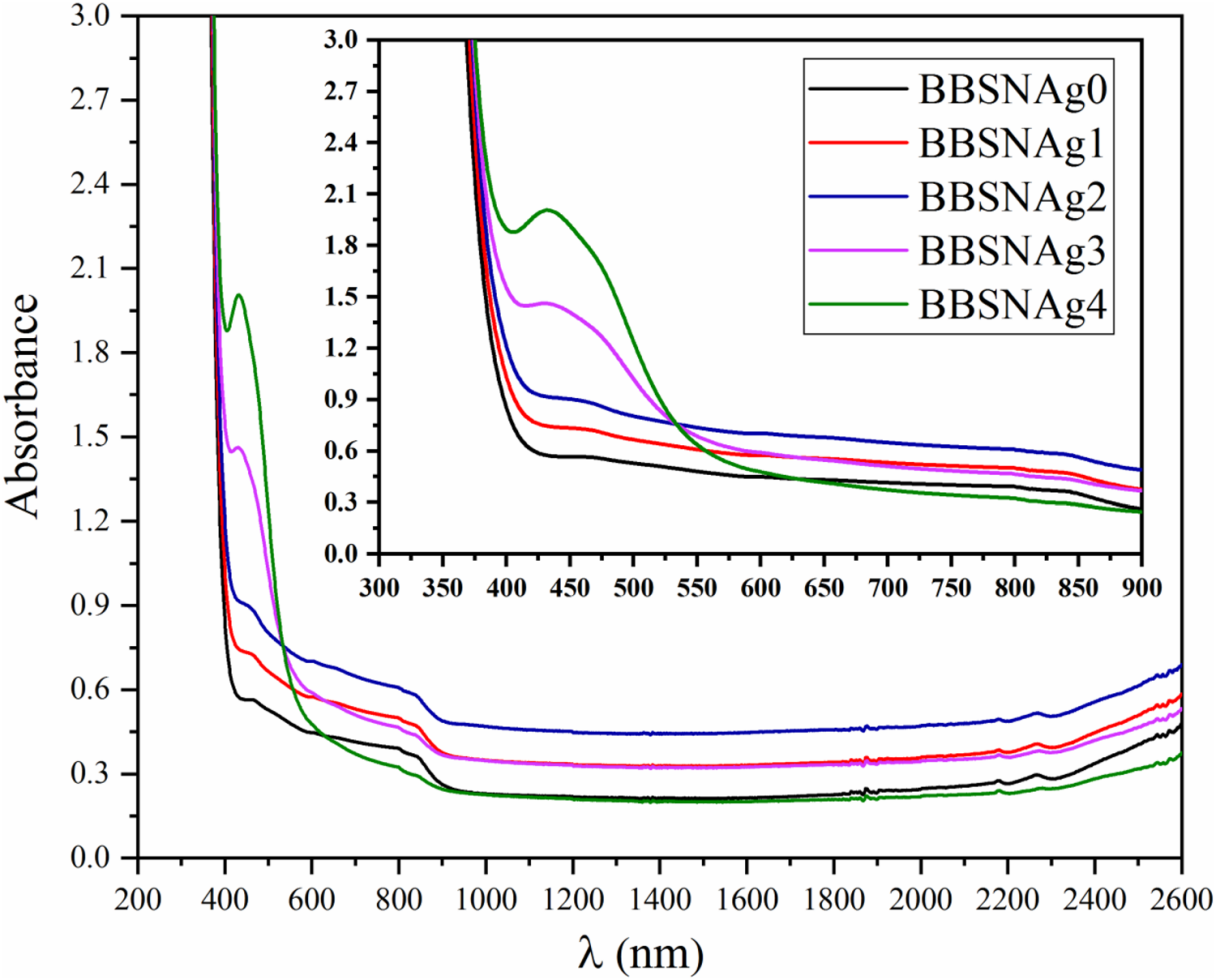
UV–visible absorption spectrum of glass samples doped with Ag_2_O.

The UV–visible absorption spectra of (55-x) B_2_O_3_ + 5Bi_2_O_3_ + 20SrF_2_ + 20Na_2_O + xAg_2_O glass samples doped with Ag_2_O are depicted in Fig. 3 in the range 190–2600 nm. The absorbance of samples improved significantly as the concentration of Ag_2_O substitution ratio increased. The absorption spectra in the range 300–900 nm are shown in the inset of Fig. 3; a broad near-visible band is evident in the spectra, centered about 423 nm. The wide near-visible band centered at 423 nm shifted towards a higher wavelength 449 nm when Ag_2_O concentration increased, owing to bandgap transitions. The unique bands of Ag_2_O were visible in these glass samples as the Ag_2_O substitution ratio increased: the peak at 435 nm is due to a ligand in the metal charge assignment transition (Ag^1+^).

The optical energy band gaps for the examined glass samples doped with Ag_2_O were estimated using Tauc's rule, which was modified by Mott and Davis, as shown in Eq. (1).

1 $$\left( {\alpha h\nu } \right)^{n} = C(h\nu - E_{g} )$$

where hν is the incident photon's energy and C is a constant. The kind of electronic transition is represented by the power (n), with n = 2 denoting a direct allowed transition and n = 0.5 denoting an indirect allowable transition.

**Figure 4:**
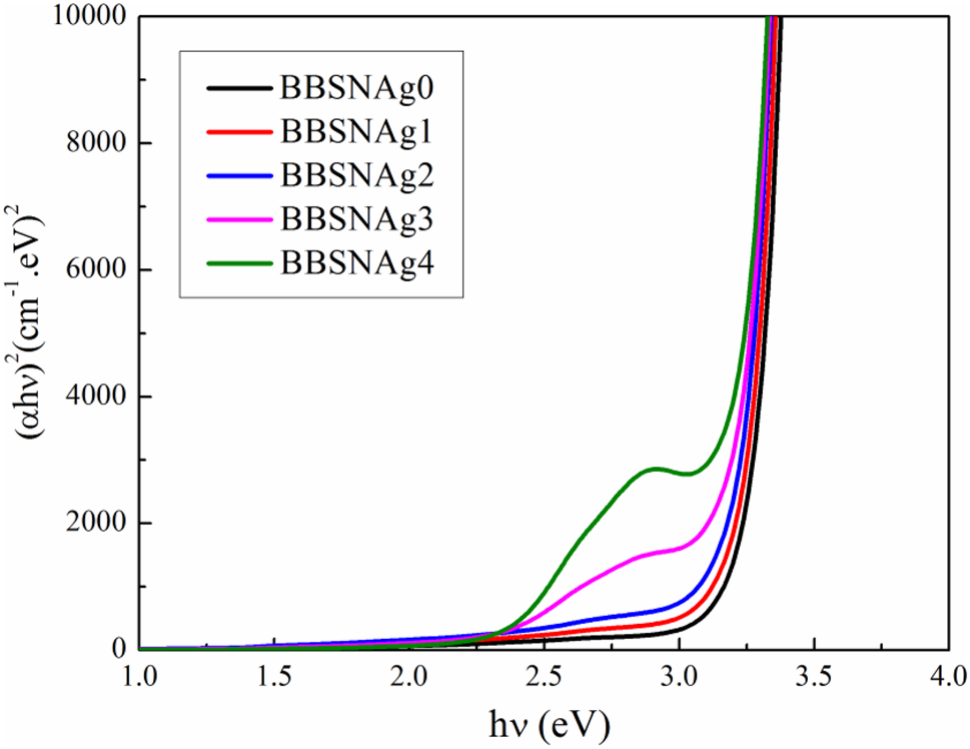
The dependence of (αhν)^2^ on (hν) for direct transitions for examined glass.

We calculated the direct bandgap energies for the produced glass samples using n = 2 and plots of (αhν)^2^ vs. hν, as shown in Fig. 4. According to the data reported in Fig. 4, glass samples' estimated direct bandgap energy decreased from 3.05 to 2.86 eV as the Ag_2_O substitution ratio increased.

**Figure 5:**
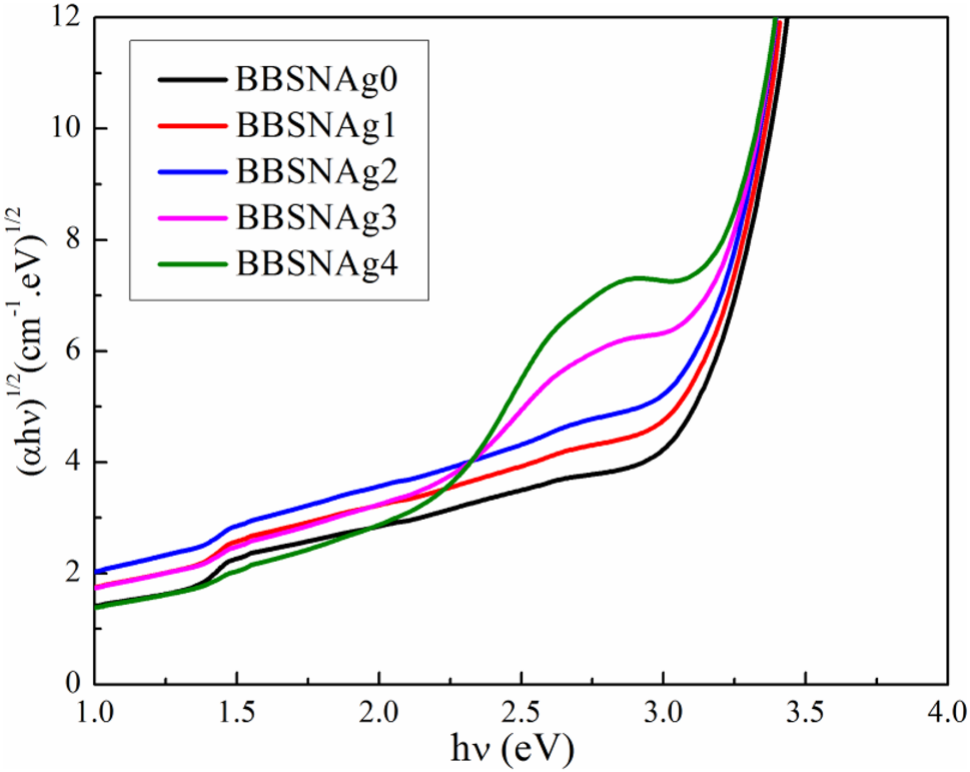
The dependence of (αhν)^1/2^ on (hν) for indirect transitions for examined glass.

From the above equation, we additionally utilized n = 1/2 to calculate the indirect bandgap energies; the indirect bandgap energies reduced from 2.68 to 2.52 eV when the Ag_2_O concentration increased, as seen in Fig. 5.

**Figure 6:**
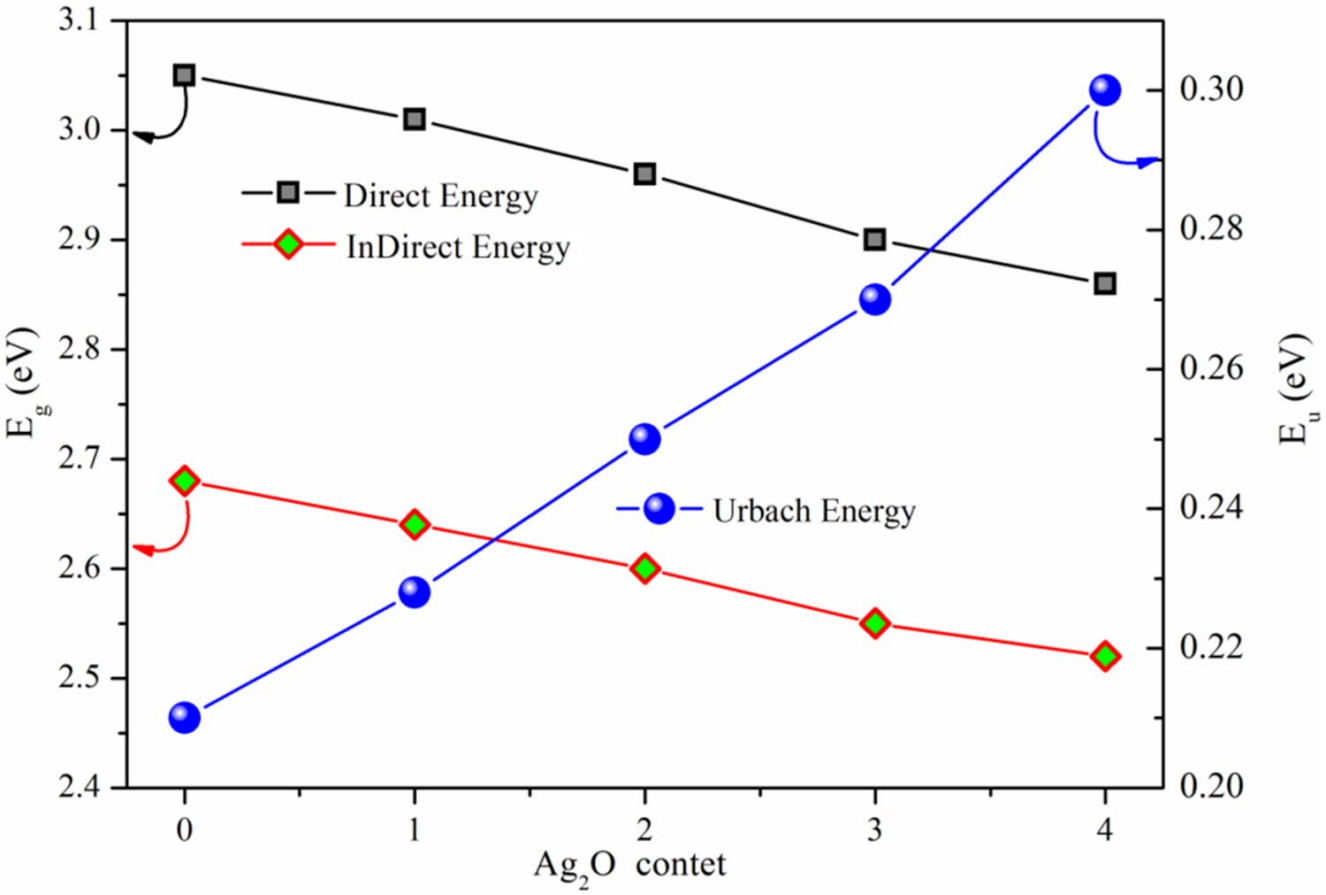
Dependence of energy gap and Urbach energy on Ag_2_O content for prepared glass samples.

The decrease of direct and indirect optical energies gap with increasing Ag_2_O substitution ratio was shown in Fig. 6. The difference between the energy band gap of Ag_2_O, which has a narrow optical gap p-type oxide semiconductor 1.3 eV^37^, and the broad bandgap energy of B_2_O_3_ (6.2 eV) might explain how the optical band gap energies decrease as the Ag_2_O substitution ratio increases.

The relationship between the absorption coefficient (α) and photon energy (hv), as stated in the given equation, is described by Urbach's empirical formula (2).

2 $$\alpha = \alpha_{0} exp\left( {\frac{hv}{{E_{u} }}} \right)$$

where *α*_*o*_ and *E*_*u*_ represent the Urbach's energy. Equation (3) can be written as expressed in Eq. 4:

3 $$ln\alpha = ln\alpha_{0} + \left( {\frac{hv}{{E_{u} }}} \right)$$

**Figure 7:**
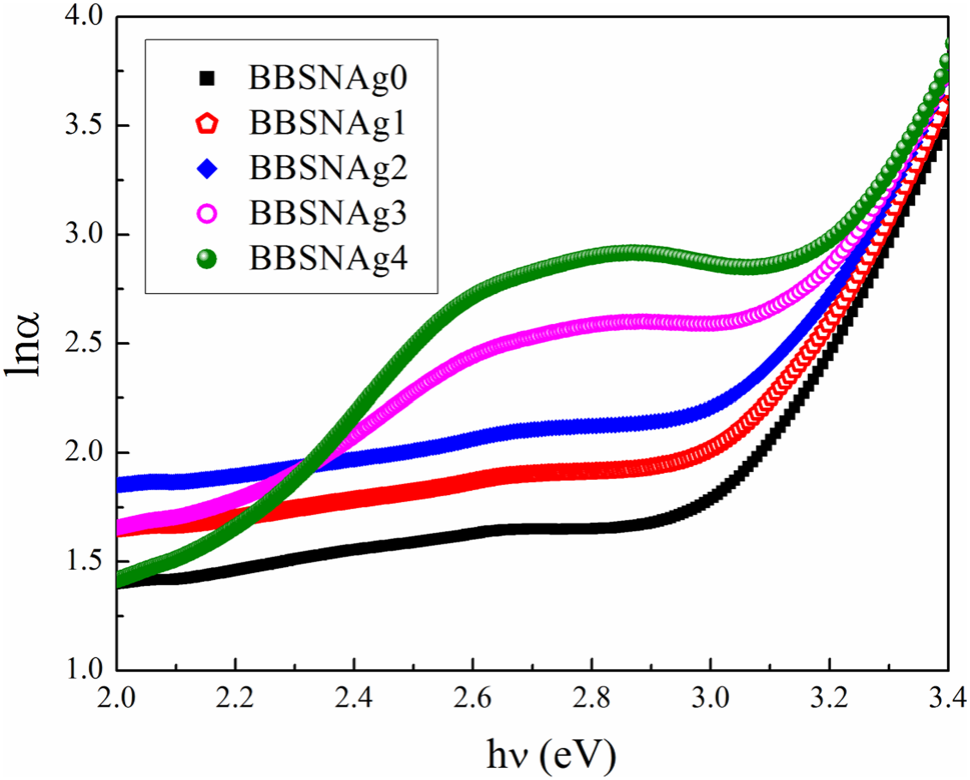
Dependence of ${\text{ln}}\left( \alpha \right)$ on the photon energy ($h\upsilon$) for prepared glasses.

As a consequence, the slope of the straight line obtained by plotting ln(α) versus (hν) could be used to calculate Urbach’s energy (E_u_). The dependence of ln(α) on the photon energy (hv) is seen in Fig. 7 for the manufactured glasses. The estimated E_u_ values were increased from 0.21 to 0.30 eV, as seen in Fig. 6; the increase in Urbach's energies implies a loss of stability and homogeneity in materials with more defects and an increase in disorder in the glass samples generated.

### Mechanical properties

**Figure 8:**
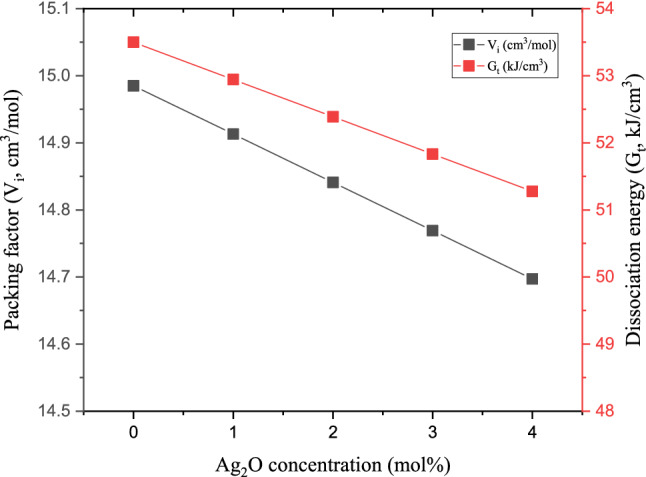
The variation of glasses’ packing factor and dissociation energy of the fabricated glasses versus the doped Ag_2_O ratio.

The previously determined density, molar mass, and molar volume were applied in the Makishima–Makenzie (M–M) model to predict the mechanical properties of the fabricated glass samples. Figure 8 illustrates a decrease in the dissociation energy (G_t_) and the packing factor (V_i_) with increasing the Ag_2_O doping ratio. The G_t_ depended mainly on the enthalpy of the reaction (heat of formation), while the packing factor depended on the ionic radius of the constituting compounds. Thus, decreasing the G_t_ values between 53.500 and 51.276 kJ/cm^3^ for the investigated glasses refers to strengthening the chemical bonds associated with increasing the Ag_2_O doping ratio. Also, this decrease in G_t_ of the fabricated glass samples is attributed to the replacement of B_2_O_3_, which has a high dissociation energy (G_i_ = 82.8 kJ/cm^3^) by the Ag_2_O with lower dissociation energy (G_i_ = 27.2 kJ/cm^3^). Figure 8 also shows a decrease in the V_i_ values from 14.985 to 14.697 cm^3^/mol, with raising the Ag_2_O between 0 and 4 mol%.

**Figure 9:**
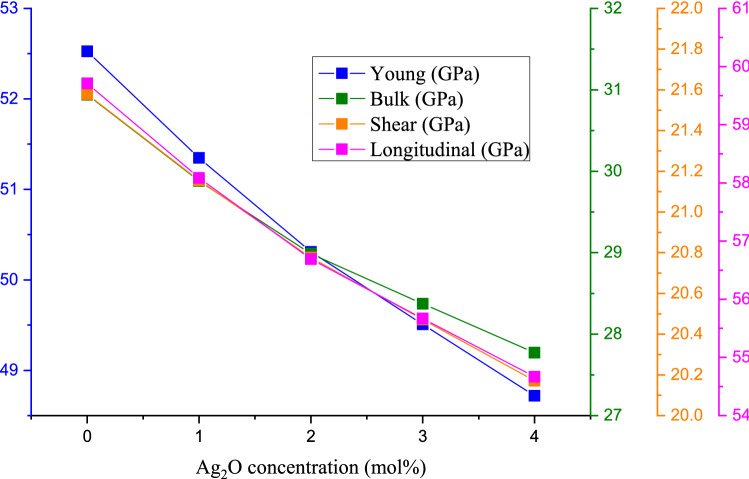
The elastic moduli (Young, shear, bulk, and longitudinal) variations at Ag_2_O doping ratio.

According to the M–M model, the mechanical modulus depended mainly on V_t_ and G_t_ values. Figure 9 shows that all the studied moduli (Young, Bulk, Shear, and Longutidunal) suffer a significant decrease with increasing the Ag_2_O concentration between 0 and 4 mol%. For example, the moduli were decreased from 52.524 to 48.719 GPa (for Young model), 30.940 to 27.774 GPa (for bulk model), from 21.578 to 20.171 GPa (for shear model), and from 59.711 to 54.669 GPa (for Longitudinal model) with increasing the Ag_2_O concentration between 0 and 4 mol%, respectively. This decrease is attributed to the packing density V_t_ reduction of the fabricated glasses, where V_t_ values decreased from 0.491 to 0.475, increasing the Ag_2_O ratio between 0 and 4 mol%. Also, the observed decrease in the mechanical module may be related to the decreases that occurred in the G_t_ values due to the replacement of B_2_O_3_ by Ag_2_O.

**Figure 10:**
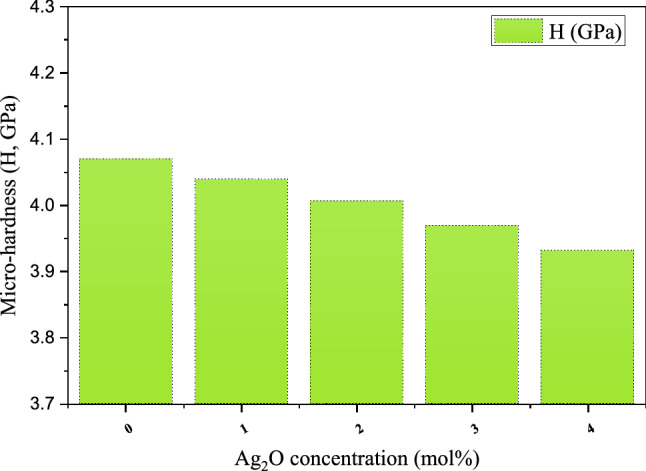
Variation of the micro-hardness of the fabricated glasses with the Ag_2_O doping ratio.

The glass microhardness estimation is very important and helpful in many shielding applications that require glasses with high microhardness. In the present study, the microhardness of the fabricated glasses was estimated based on the calculated values of the Poisson ratio, which is illustrated in Table 2. Figure 10 illustrates that the insertion of the Ag_2_O on the Bi_2_O_3_–Na_2_O–Sr_2_F–B_2_O_3_ glass system increases the microhardness of the fabricated samples. The microhardness decreased from 4.070 to 3.931 GPa, increasing the Ag_2_O doping ratio between 0 and 4 mol%, respectively.

**Table 2:** The mechanical properties of the fabricated BBSNAg glass samples.

	Packing density (V_t_)	Poisson ratio	Longitudinal velocity (V_l_, m/s)	Shear velocity (V_s_, m/s)	Softening temperature	Fractal bond conductivity
BBSNAg0	0.491	0.217	4288.580	2578.071	399.869	2.790
BBSNAg1	0.485	0.214	4211.215	2541.431	391.468	2.832
BBSNAg2	0.480	0.211	4137.569	2504.823	382.191	2.867
BBSNAg3	0.478	0.209	4069.116	2467.583	371.104	2.886
BBSNAg4	0.475	0.208	4002.120	2431.002	360.322	2.905

### Shielding properties

**Figure 11:**
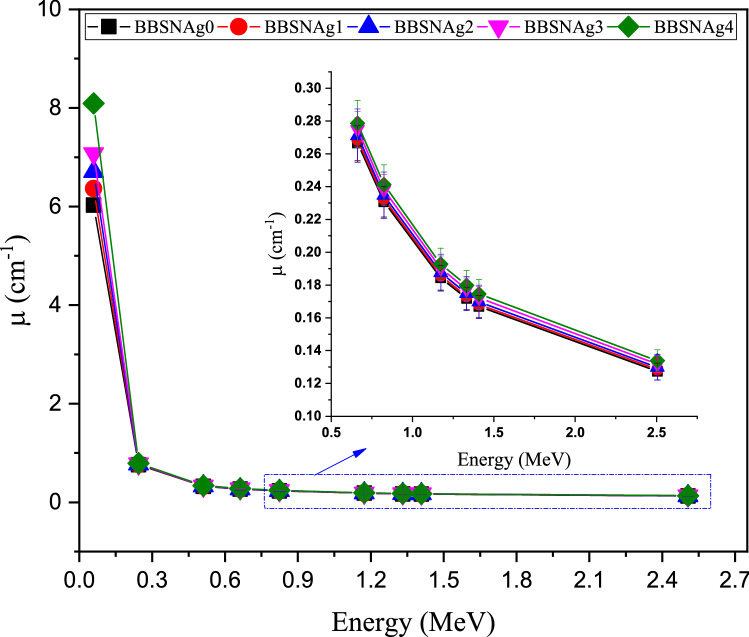
Variation of the linear attenuation coefficient versus the gamma-ray energy in the interval between 0.059 and 2.506 MeV.

A code based on the Monte Carlo simulation was utilized to estimate the shielding capacity for the intermediate gamma-ray energy photons between 0.059 and 2.506 MeV. At gamma photons with an energy of 0.059 MeV, the linear attenuation coefficient (µ, cm^−1^) reaches the maximum values for the fabricated BBSNAg samples. At the mentioned energy, Fig. 11 refers to the µ values varied between 6.029 and 8.091 cm^−1^ for samples BBSAg0 and BBSNAg4, respectively. The energy of 0.059 MeV was included in the photoelectric interaction (PE) region. Also, the PE cross-section inversely varied with the E^3.5^, so the cross-section of interaction increases to a maximum at low gamma-ray energy, which increases the µ of the samples to maximum values^38,39^. In subfigure 11, the gamma-ray energy varied between 0.244 and 1.408 MeV, associated with a decrease in the µ values for all studied samples. The recorded decrease in the µ values is due to the Compton scattering (CS), which is popular in the mentioned energy interval. For the CS interaction, the cross-section of interaction varied inversely with E. thus, the cross-section of interaction decreases with raising the incident photon energy. The net results are a decrease in the number of collisions between the photons and the material atoms associated with an exponential decrease in the µ values. In this energy interval the average µ values recorded for the fabricated samples are 0.301, 0.303, 0.306, 0.310, and 0.314 cm^−1^ for glass samples BBSNAg0, BBSNAg1, BBSNAg2, BBSNAg3, and BBSNAg4, respectively. The lowest µ values were observed at 2.506 MeV among the selected energy range. They varied between 0.127 and 0.134 for samples BBSAg0 and BBSNAg4.

The influence of Ag_2_O doping on the attenuation capacity of the fabricated glass BBSNAg glass samples was also studied. The data presented in Fig. 11 showed that increasing the doping ratio between 0 and 4 mol% causes a significant increase in the µ values of the fabricated BBSNAg glasses. The sample BBSNAg0 without Ag_2_O doping has µ values between 6.029 and 0.127 cm^−1^. These values improved to 8.091 and 0.134 cm^−1^ for BBSNAg4 at gamma-ray energy 0.059 and 2.506 MeV. The mentioned results show an increase in the µ values due to increasing the Ag_2_O doping ratio. Increasing the Ag_2_O doping ratio causes an increase in the molar mass and electron density of the fabricated glasses, which causes an increase in the number of collisions between gamma photons and the glass atoms. Thus, the deposited energy inside the glass increases associated with an increase in the µ values.

**Figure 12:**
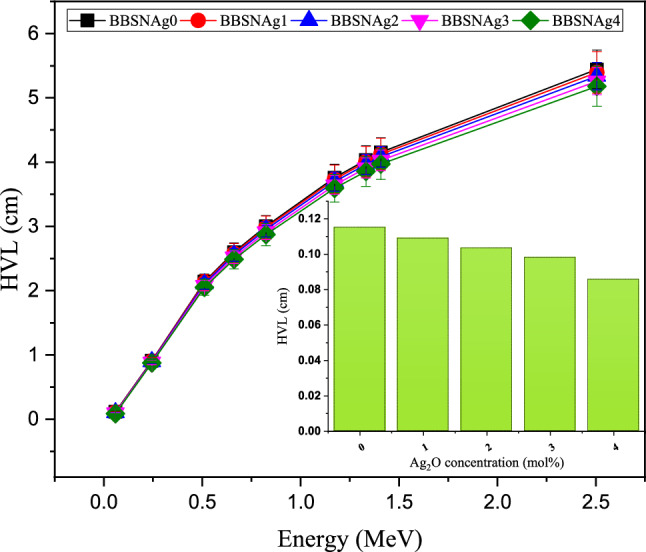
Variation of the half-value layer of the fabricated glasses at various gamma-ray energies.

The half-value layer is a thickness of the shielding material, which has the ability to reduce the incident activity of the source to half. In the energy interval between 0.059 and 2.506 MeV, the thinner HVL was observed at 0.059 MeV, where the HVL varied between 0.115, 0.109, 0.103, 0.098, and 0.086 cm for the fabricated samples BBSNAg0, BBSNAg1, BBSNAg2, BBSNAg3, and BBSNAg4, respectively. The HVL of the fabricated BBSNAg glasses increases and reaches a maximum at a gamma photon energy of 2.506 MeV, where the maximum values in the present study are 5.442, 5.395, 5.337, 5.259, and 5.180 cm. In the studied energy range, the average HVL is 2.906, 2.884, 2.856, 2.815, and 2.775 cm for the investigated glass samples BBSNAg0, BBSNAg1, BBSNAg2, BBSNAg3, and BBSNAg4, respectively. The increase in the HVL values is related to the inverse proportionality of the HVL with the µ values, where µ ≈ 0.693/HVL. Hence the µ values for all fabricated glass samples decrease with the incident photon energy, so the HVL exceeds with raising the photon energy. Also, Fig. 12 illustrates that doping of bismuth sodium-strontium borate glasses with Ag_2_O enhances the shielding capacity of the fabricated glasses. Thus, the HVL was reduced by a factor of 5.276, 10.123, 14.790, and 25.485% with raising the doping ratio from 1 to 4 mol%, respectively, at gamma-ray energy of 0.059 MeV.

**Figure 13:**
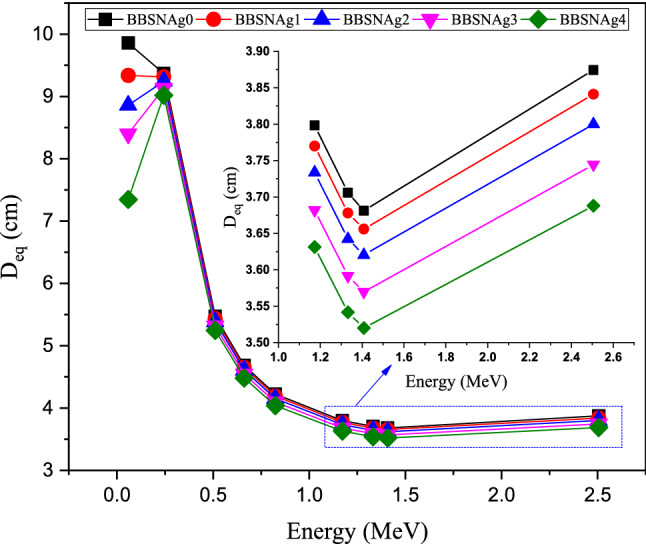
Variation of the D_eq_ (cm) of the fabricated glasses at various gamma-ray energies.

The thickness equivalent (D_eq_, cm) is calculated to estimate the shielding capacity of the fabricated BBSNAg samples compared to the pure lead element. Figure 13 illustrates the thickness of the fabricated glasses equivalent to 1 cm of the lead. Figure 13 shows an increase in the D_eq_ values in the energy interval between 0.059 and 0.244 MeV. The increase in D_eq_ values in this region is related to the huge increase in the lead µ value around 0.088 MeV (due to the lead K-absorption edges). In the mentioned interval, the highest D_eq_ was achieved for samples BBSNAg0 and BBSNAg1 because they are the lowest linear attenuation coefficient glasses in the present study (due to the lowest content in Ag). With increasing the Ag_2_O concentration in the fabricated glasses, the D_eq_ decreases regard. Thus, (D_eq_) _BBSNAg2_ > (Deq) _BBSNAg3_ > (D_eq_) _BBSNAg4_. At this energy interval, the D_eq_ has an average value of 9.613, 9.327, 9.052, 8.765, and 8.182 cm for glass samples BBSNAg0 and BBSNAg1 BBSNAg2, BBSNAg3, and BBSNAg4, respectively. After that, the D_eq_ decreases rapidly above 0.244 MeV. This slight decrease of the glasses’ µ values compared to Pb’s µ values. In the energy interval between the 0.244 and 1.408 MeV, the average D_eq_ varied between 4.261, 4.230, 4.190, 4.133, and 4.077 cm for glass samples BBSNAg0, BBSNAg1, BBSNAg2, BBSNAg3, and BBSNAg4, respectively. This means that in the CS interaction region, the shielding capacity of the fabricated glasses is about around 25% of the pure lead. Also, Fig. 13 illustrates that in the pair production (PP) interaction region (i.e., 2.506 MeV), the increase in the Pb’s µ values is higher than the increase observed for the fabricated glasses. This is because the PP cross-section is directly varied with Z^2^_,_ so the Pb’s µ values rise more elevated than that of the fabricated glasses with lower adequate atomic numbers.

**Figure 14:**
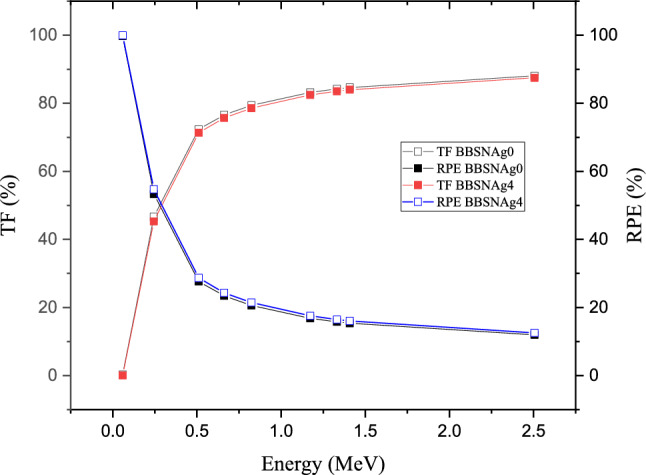
Variation of the Transmission factor (TF, %) and the radiation protection efficiency (RPE, %) versus gamma-ray energies for samples BBSNAg0 and BBSNAg4.

The transmission factor (TF, %) and the radiation protection (RPE, %) are two inversely proportional factors, where the TF is a measure for the photons that can penetrate the shielding material thickness. At the same time, the RPE measures the amount of energy deposited inside the shielding thickness. Figure 14 illustrates the variation of the TF and RPE against the applied gamma-ray photon energy. The figure shows that the TF and RPE varied through a variation in the photon energy or the amount of Ag_2_O doping ratio. Figure 14 shows that the TF has the highest values at high gamma-ray energy due to the high penetration power posses by the photons where the penetration power increases with raising the photon energy, so the gamma photons travel along its path length with a low number of collisions between the photon and its surrounding atoms. Thus, the number of photons passing the shielding thickness is maximum (i.e., TF is maximum) while the energy deposited inside the shielding material reaches the minimum values (i.e., RPE is minimum). For example, at the gamma-ray energy of 2.506 MeV, the TF varied between 88.040 and 87.475 while the RPE varied between 11.96 and 12.53% for the glass samples BBSNAg0 and BBSNAg4, respectively.

The second ratio of the dopant compound also significantly influences both TF and RPE values. For example, at gamma-ray energy of 0.059 MeV, the TF values decrease from 0.172 to 0.031%, while the RPE is increased from 99.760 to 99.970% with increasing the Ag_2_O ratio between 0 and 4 mol%, respectively. Although, as previously illustrated in Fig. 2, increasing the dopant Ag_2_O ratio causes a significant increase in the density of the fabricated glass samples from 3.247 to 3.413 g/cm^3^, this increase in the density was associated with an increase in the resistance and collisions between the incident photon and the glass atoms and electrons. As a result, the number of photons that can pass the shielding thickness decreases (i.e., TF decreases), and the energy deposited inside the shielding thickness increases (i.e., RPE increases).

**Figure 15:**
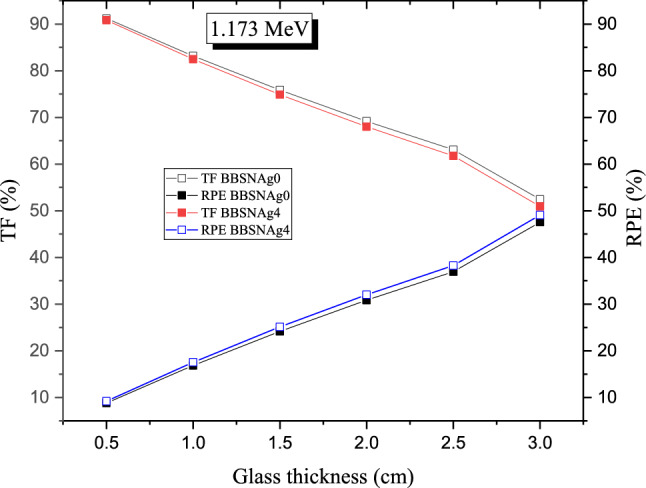
Variation of the transmission factor (TF, %) and the radiation protection efficiency (RPE, %) versus the glass thickness for samples BBSNAg0 and BBSNAg4.

Both calculated TF and RPE are affected by the glass thickness, as presented in Fig. 15. The TF decreases associated with an increase in the RPE values when the fabricated BBSNAg glass thickness increases from 0.5 to 3 cm. This behavior is related to the path length of gamma photons inside the fabricated samples. Increasing the glass thickness causes an increase in the path length of gamma photons inside the fabricated glass samples. Thus, the number of photons-glass atoms collision increases associated with an increase in the energy deposited inside the glass layer. As a result, the RPE increases with growing the fabricated BBSNAg glass's thickness, which is associated with a decrease in the number of photons penetrating the glass (i.e., TF decreases). For example, at a gamma photon energy of 1.173 MeV, the RPE increases from 9.192 to 49.08 1% (for glass sample BBSNAg4) and from 8.806 to 47.547% (for glass sample BBSNAg0) while the TF decreases from 91.194 to 52.454% (for glass sample BBSNAg0) and from 90.808 to 50.919% (for glass sample BBSNAg4) when the glass thickness increases from 0.5 to 3 cm, respectively.

## Conclusion

In the present work, the partial substitution of B_2_O_3_ by Ag_2_O causes significant changes in the density, mechanical, optical, and radiation shielding properties of (55-x) B_2_O_3_ + 5 Bi_2_O_3_ + 20SrF_2_ + 20Na_2_O + xAg_2_O glass system where x value varied between 0 and 4 mol%. The glass density increases from 3.247 to 3.413 g/cm^3^ with raising the Ag_2_O concentration between 0 and 4 mol%. This increase in the density of the samples was associated with decreasing in the mechanical moduli and microhardness, where the mechanical moduli decreased from 52.524 to 48.719 GPa (for Young’s model), from 30.940 to 27.774 GPa (for bulk model), from 21.578 to 20.171 GPa (for shear model), and from 59.711 to 54.669 GPa (for longitudinal model). Moreover, the microhardness decreased from 4.70 to 3.931 GPa. Concerning the optical properties, the direct bandgap energy reduced from 3.05 to 2.86 eV, and the indirect bandgap energy reduced from 2.68 to 2.52 eV. On the other hand, the glasses density positively affects the gamma-ray shielding capacity enhancement. The linear attenuation coefficient was enhanced from 6.029 to 8.091 cm^−1^, raising the Ag_2_O concentration between 0 and 4 mol%, at gamma-ray energy of 0.059 MeV, respectively. The enhancement in the linear attenuation coefficient of the fabricated samples was associated with an increase in the radiation protection efficiency and a decrease in the transmission factor and half-value thickness of the evaluated glass samples. Based on the results reported in this article, a sample with 4 mol% of the Ag_2_O compound has the best shielding properties where the linear attenuation coefficient varied between 8.091 and 0.134 when the gamma-ray energy raised from 0.059 to 2.506 MeV, respectively.
